# *Plastrum testudinis* Ameliorates Oxidative Stress in Nucleus Pulposus Cells via Downregulating the TNF-α Signaling Pathway

**DOI:** 10.3390/ph16101482

**Published:** 2023-10-17

**Authors:** Peng Zhang, Jiahui He, Yanchi Gan, Qi Shang, Honglin Chen, Wenhua Zhao, Gengyang Shen, Xiaobing Jiang, Hui Ren

**Affiliations:** 1 Guangzhou University of Chinese Medicine, Guangzhou 510405, China; 20201120134@stu.gzucm.edu.cn (P.Z.); ganyanchi@stu.gzucm.edu.cn (Y.G.); 20181101008@stu.gzucm.edu.cn (Q.S.); spinedrchl@163.com (H.C.); 2 The Affiliated TCM Hospital of Guangzhou Medical University, Guangzhou 510130, China; 20181101062@stu.gzucm.edu.cn; 3 Department of Spinal Surgery, The Second Affiliated Hospital of Guangzhou Medical University, Guangzhou 510260, China; 13539961409@163.com (W.Z.); 15920198161@163.com (G.S.)

**Keywords:** intervertebral disc degeneration, *Plastrum testudinis*, bioinformatic analysis, experimental validation, oxidative stress, TNF-α signaling pathway

## Abstract

**Background** *Plastrum testudinis* (PT), a widely used traditional Chinese medicine, exerts protective effects against bone diseases such as intervertebral disc degeneration (IDD). Despite its effectiveness, the molecular mechanisms underlying the effects of PT on IDD remain unclear. **Methods** In this study, we used a comprehensive strategy combining bioinformatic analysis with experimental verification to investigate the possible molecular mechanisms of PT against IDD. We retrieved targets for PT and IDD, and then used their overlapped targets for protein–protein interaction (PPI) analysis. In addition, we used Gene Ontology (GO) and Kyoto Encyclopedia of Genes and Genomes (KEGG) enrichment analyses to investigate the anti-IDD mechanisms of PT. Moreover, in vivo and in vitro experiment validations including hematoxylin–eosin (HE) and safranine O-green staining, senescence-associated β-galactosidase (SA-β-gal) assay, cell immunofluorescence staining, intracellular ROS measurement and Western blot analysis were performed to verify bioinformatics findings. **Results** We identified 342 and 872 PT- and IDD-related targets (32 overlapping targets). GO enrichment analysis yielded 450 terms related to oxidative stress and inflammatory response regulation. KEGG analysis identified 48 signaling pathways, 10 of which were significant; the TNF-α signaling pathway had the highest *p*-value, and prostaglandin G/H synthase 2 (*PTGS2*), endothelin-1 (*EDN1*), TNF-α, *JUN* and *FOS* were enriched in this pathway. Histopathological results and safranin O/green staining demonstrated that PT attenuated IDD, and SA-β-gal assay showed that PT ameliorated nucleus pulposus cell (NPC) senescence. An ROS probe was adopted to confirm the protective effect of PT against oxidative stress. Western blot analyses confirmed that PT downregulated the protein expression of *PTGS2*, *EDN1*, TNF-α, *JUN* and *FOS* in the TNF-α signaling pathway as well as cellular senescence marker p16, proinflammatory cytokine interleukin-6 (*IL6*), while PT upregulated the expression of NPC-specific markers including *COL2A1* and *ACAN* in a concentration-dependent manner. **Conclusions** To the best of our knowledge, this study is the first to report that PT alleviates IDD by downregulating the protein expression of *PTGS2*, *EDN1*, TNF-α, *JUN* and *FOS* in the TNF-α signaling pathway and upregulating that of *COL2A1* and *ACAN*, thus suppressing inflammatory responses and oxidative stress in NPCs.

## 1. Introduction

As a chronic inflammatory disease, intervertebral disc degeneration (IDD) has an increasing prevalence with the aging of society [1]. IDD is an important factor in lower back pain with a prevalence of 80% among adults without effective therapeutic treatment [2,3], which is a common clinical condition that causes long-term pain and, potentially, the inability to work; it seriously affects quality of life, and imposes a major financial and social burden on families and the economy [4,5]. IDD is closely related to age, load-bearing, trauma, genetics, inflammation, and tissue injury induced by oxidative stress [6]. An existing study has confirmed that IDD is triggered mainly by oxidative stress and inflammatory infiltration, which is characterized by apoptosis of nucleus pulposus cells and extracellular matrix degradation [7].

Many inflammatory factors in degenerated discs, such as tumor necrosis factor-α (TNF-α) [8], interleukin-1β (IL-1β) [9], and matrix metalloproteinases (MMPs) [10], trigger the production of reactive oxygen species (ROS). TNF-α is a potent inflammatory cytokine with powerful proinflammatory activities associated with the secretion of multiple proinflammatory mediators. TNF-α is upregulated in IDD and closely linked to numerous associated pathological processes, including oxidative stress, inflammation, cellular senescence, and apoptosis [11]. Therefore, there is the potential for anti-TNF-α therapies to reduce oxidative stress and inflammatory responses, thus alleviating IDD.

In recent years, the protective effects of traditional Chinese medicine against IDD have received increasing attention [12]. According to the Chinese Pharmacopoeia (2015), *Plastrum testudinis* (PT; Testudinis Carapax et Plastrum), is organic in nature from the plastron and carapace of *Chinemys reevesii* (Gray), which is one of the most widely used traditional Chinese medicines for treating skeletal disorders [13]. Existing evidence shows that PT acts as an anti-inflammatory drug with significant antioxidant property, containing bioactive components such as steroids (cholesterol, 4-cholesten-3-one and cholesterol myristate), fatty acids (stearic acid and palmitic acid), and esters (methyl palmitate, ethyl palmitate, methyl stearate, and ethyl stearate), which could promote the proliferation and growth of bone cells [14]. Steroids are involved in osteoblast proliferation via the NF-κB signaling pathway [15], while fatty acids can promote alkaline phosphatase (ALP) activity [16]. Palmitic acid and stearic acid have been reported to regulate inflammatory cytokines and reduce inflammation via suppressing ROS-activated p38 MAPK/ERK-Akt and NF-κB activity [17,18,19]. Cholesterol and its derived steroids play a significant role in regulating osteoblast differentiation [20]. Our previous studies have reported that PT can reverse the imbalance between bone formation and resorption by regulating osteoblastic and osteoclastic markers including OPG, RUNX2, RANKL, RANK and CTSK [21,22]. In terms of bone formation, PT can also promote osteogenic differentiation by upregulating the expression of the p38 MAPK, and inhibiting the expression of TRAF6 [23] and the TNFR2/PI3K/AKT Signaling Pathway [24]. In terms of bone resorption, PT can suppress osteoclastic differentiation via the NF-κB signaling pathway [22]. Moreover, a previous study showed that PT exerted anti-inflammatory and proliferative effects on annulus fibrosus (AF) cells, and thus is an effective treatment for IDD induced by degeneration and inflammation of AF tissue [25]. However, due to the lack of relevant research, the molecular mechanisms underlying the therapeutic effects of PT on IDD remain unclear.

In this study, we carried out a comprehensive bioinformatic analysis and experimental verification of various targets and pathways associated with the anti-IDD properties of PT.

## 2. Results

### 2.1. PT-Associated Components and Target Proteins

From the BATMAN-TCM database, we obtained six bioactive components (threonine, aspartic acid, calcium carbonate, methionine, leucine, and phenylalanine) and 342 targets for PT.

### 2.2. IDD-Related Target Proteins and PPI Network Construction

**Figure 1:**
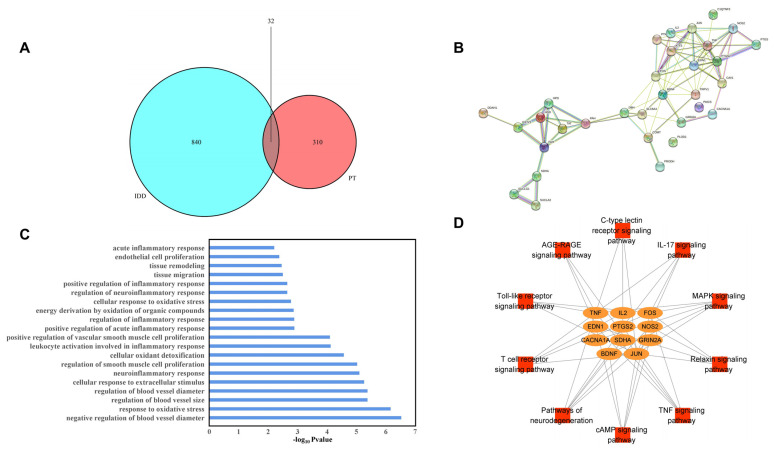
Venn diagram of overlapping (between PT and IDD) targets (**A**), PPI network of potential targets (**B**), GO BP enrichment results (**C**), and the pathway–target network (**D**).

We identified 872 IDD-related target proteins. There were 32 overlapping target proteins (OTPs, between PT- and IDD-associated targets) (Table 1 and Figure 1A). The PPI network of OTPs is plotted in Figure 1B.

### 2.3. GO Enrichment Analysis

A total of 450 biological process (BP) GO terms were identified (*p* < 0.05). Twenty terms were mainly associated with oxidative stress, the regulation of inflammation, positive regulation of ROS metabolism, the cellular response to extracellular stimuli, regulation of blood vessel size, tissue migration, and remodeling. Therefore, all of these processes are closely associated with IDD, as shown in Figure 1C.

### 2.4. KEGG Pathway Analysis

Of the 48 KEGG signaling pathways identified, 10 were significant (*p* < 0.05), as shown in Table 2. We performed network visualization (Figure 1D) and the results showed that the TNF-α signaling pathway had the highest *p*-value; PTGS2, EDN1, TNF-α, JUN and FOS, which may perform essential functions in the metabolic process through which PT acts against IDD, were enriched in this pathway.

### 2.5. CCK-8 Analysis and SA-β-Gal Activity Assessment

**Figure 2:**
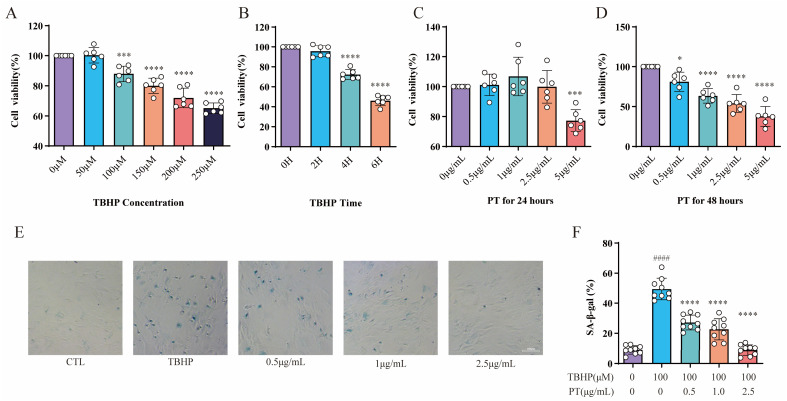
**CCK-8 and SA-β-gal activity assay results**. Cell viability was analyzed for NPCs treated with TBHP (concentration gradient = 0, 50, 100, 150, 200, and 250 μM) for 2, 4, and 6 h (**A**,**B**), or with PT (concentration gradient = 0, 0.5, 1, 2.5, and 5 μg/mL) for 24 and 48 h (**C**,**D**). * *p* < 0.05, *** *p* < 0.001, and **** *p* < 0.0001 vs. 0 μM or 0 h. (**E**) Representative SA-β-gal staining images of NPCs treated with TBHP, with or without PT. (**F**) Histograms of SA-β-gal activity in the three groups. Scale = 100 μm. Data are the mean ± standard deviation. #### *p* < 0.0001 vs. CTL group; **** *p* < 0.0001 vs. 100 μM TBHP group.

The assay concentrations of TBHP were 0 (for CTL), 50, 100, 150, 200 and 250 μmol/L (μM). The THBP concentration at 100 μM after 4 h suppressed the proliferation of NPCs; in turn, this induced inflammation and oxidative stress, thereby contributing to the pathogenesis of IDD. Thus, a THBP concentration of 100 μM (after 4 h) was selected for subsequent experiments (Figure 2A,B). The assay concentrations of PT were 0 (for CTL), 0.5, 1, 2.5, and 5 μg/L. The CCK-8 results showed that PT exerted no cytotoxic effect on NPCs at concentrations ≤ 2.5 μg/mL after 24 h, and neither inhibited nor promoted the proliferation of NPCs; therefore, this concentration was used in subsequent experiments (Figure 2C,D). The results showed that the SA-β-Gal-positive NPCs in the TBHP group increased remarkably relative to the CTL group. However, the addition of PT significantly reduced SA-β-Gal staining-positive NPCs in a concentration-dependent manner from 0.5 to 2.5 μg/mL (Figure 2E,F).

### 2.6. Expression of PTGS2, FOS, JUN, EDN1 and TNF-α in NPCs

**Figure 3:**
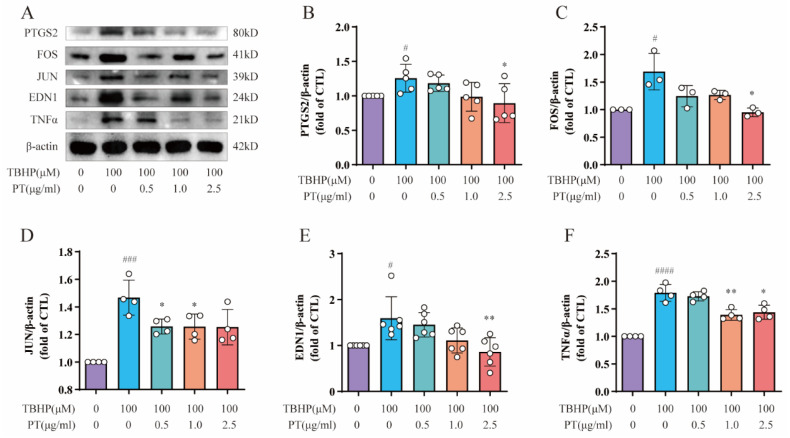
**Expression of PTGS2, FOS, JUN, EDN1 and TNF-α in NPCs**. (**A**) Expression of PTGS2, FOS, JUN, EDN1 and TNF proteins in NPCs treated with TBHP, with or without PT. (**B**–**F**) Expression of PTGS2, FOS, JUN, EDN1 and TNF proteins relative to β-actin in NPCs treated with TBHP, with or without PT. Data are the mean ± standard deviation. # *p* < 0.05, ### *p* < 0.001, #### *p* < 0.0001 vs. the CTL group; * *p* < 0.05, ** *p* < 0.01 vs. the TBHP group treated with 100 μM TBHP.

We conducted Western blotting to detect the protein expression of PTGS2, FOS, JUN, EDN1 and TNF-α, all of which are enriched in the TNF-α signaling pathway, in NPCs. As Figure 3 demonstrates, the protein expression of PTGS2, FOS, JUN, EDN1 and TNF was upregulated after TBHP treatment, unlike in the CTL, while PT (0.5–2.5 μg/mL) significantly downregulated protein expression.

### 2.7. Expression of COL2A1 and ACAN in NPCs

**Figure 4:**
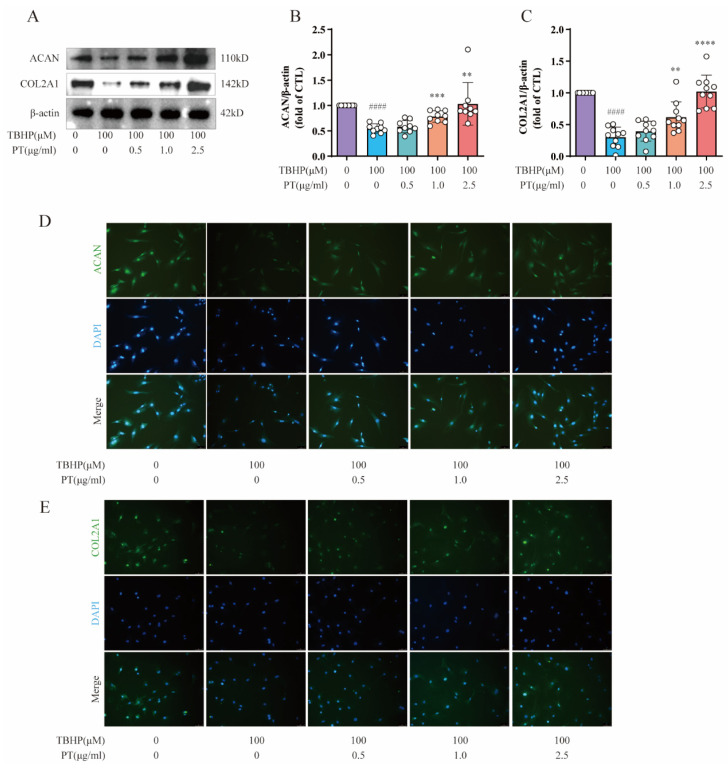
**Expression of ACAN and COL2A1 in NPCs**. (**A**) Expression of ACAN and COL2A1 proteins in NPCs treated with TBHP, with or without PT. (**B**,**C**) Expression of ACAN and COL2A1 proteins relative to β-actin in NPCs treated with TBHP, with or without PT. (**D**) Representative immunofluorescence images of ACAN in NPCs photographed by fluorescence microscopy (scale bar = 100 μm). (**E**) Representative immunofluorescence images of COL2A1 in NPCs photographed by fluorescence microscopy (scale bar = 100 μm). Data are the mean ± standard deviation. #### *p* < 0.0001 vs. CTL group; ** *p* < 0.01, *** *p* < 0.001, and **** *p* < 0.0001 vs. 100 μM TBHP group.

Importantly, NPC-specific anabolic genes, including COL2A1 and ACAN, have important functions in the pathological process of IDD. Therefore, we also detected the protein expression of COL2A1 and ACAN using Western blot and immunofluorescence assays. As Figure 4 shows, protein expression and fluorescence intensity of COL2A1 and ACAN was reduced after TBHP treatment, unlike in the CTL, while PT (0.5–2.5 μg/mL) significantly upregulated their protein expression and fluorescence intensity.

### 2.8. Detection of p16, IL6, TNF-α, and ROS Levels in NPCs

**Figure 5:**
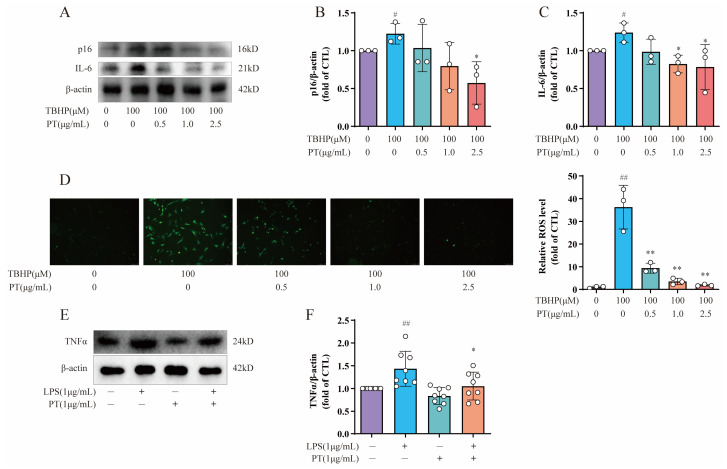
**Detection of p16, IL6, TNF-α, and ROS levels in NPCs**. (**A**) Expression of p16 and IL-6 proteins in NPCs treated with TBHP, with or without PT. (**B**,**C**) Expression of p16 and IL-6 proteins relative to β-actin in NPCs treated with TBHP, with or without PT. (**D**) ROS production in NPCs treated with TBHP, with or without PT. (**E**) Expression of TNF-α protein in NPCs treated with LPS, with or without PT. (**F**) Expression of TNF-α protein relative to β-actin in NPCs treated with LPS, with or without PT. Data are the mean ± standard deviation. # *p* < 0.05, ## *p* < 0.01 vs. CTL group; * *p* < 0.05, ** *p* < 0.01 vs. 100 μM TBHP or 1 μg/mL LPS group.

To further explore the roles of cellular senescence, inflammation, and oxidative stress during IDD, we also selected cellular senescence marker p16, inflammatory factor IL-6, and an ROS probe DCFH-DA for subsequent testing. As Figure 5A–C demonstrates, the protein expression levels of p16 and IL-6 were upregulated after the TBHP treatment relative to the CTL, while PT (0.5–2.5 μg/mL) significantly downregulated protein expression; this verified the protective effect of PT against cellular senescence and inflammation. Notably, intracellular ROS levels in TBHP-induced NPCs were detected by ROS probe DCFH-DA, using fluorescence intensity to monitor the ROS levels. Fluorescence microscopy showed that the green fluorescence was brighter in cells treated with 100 μM of TBHP after 4 h than that in CTL cells, and this effect was reversed by PT in a concentration-dependent manner from 0.5–2.5 μg/mL (Figure 5D). In addition, to verify the protective effect of PT via blocking the TNF-α signaling pathway, we used lipopolysaccharide (LPS; L2880) purchased from Sigma-Aldrich (St. Louis, MO, USA), which acts as an agonist on the TNF-α signaling pathway. The results confirmed that LPS upregulated TNF-α expression, whereas PT downregulated TNF-α expression by blocking the TNF-α signaling pathway (Figure 5E,F).

### 2.9. Histological Staining Analysis

**Figure 6:**
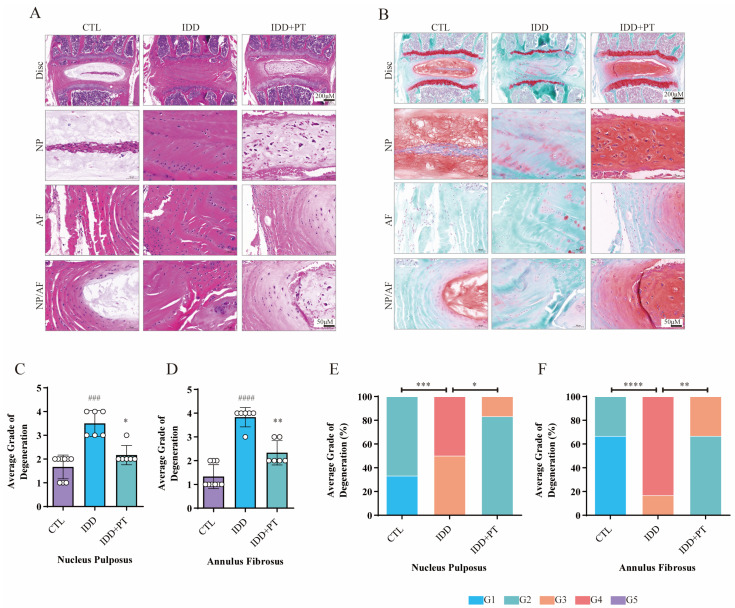
**Histological staining results**. Three-month-old wild-type C57BL/6 mice were randomly divided into three groups: CTL (*n* = 9), IDD (*n* = 6), and IDD+PT (*n* = 6). Representative HE (**A**) and safranin O/green-stained (**B**) images of intervertebral discs, and histograms generated via histological assessment (**C**–**F**) of the three groups. Scale bar = 200 μm. G1–G5 correspond to gradually increasing degeneration grades. ### *p* < 0.001, #### *p* < 0.0001 vs. CTL group; * *p* < 0.05, ** *p* < 0.01, *** *p* < 0.001, and **** *p* < 0.0001 vs. IDD group.

Figure 6 shows the results of HE and safranin O/green staining. It is clear that the intervertebral discs in the IDD group exhibited greater degeneration compared to the CTL group, such as disappearance of the nucleus pulposus, which was replaced by disorganized AF; further, the orderly arrangement of the AF was destroyed and the endplate partly disappeared. This indicates successful establishment of an IDD model of naturally aging mice. Notably, treatment with PT protected the structure of intervertebral discs. The in vivo results confirmed that PT can suppress IDD progression.

## 3. Discussion

PT, which is a widely used traditional Chinese medicine, has protective effects against bone disorders such as osteoporosis and fracture [13,22,23]. In addition, PT may be beneficial for IDD via its anti-inflammatory and cell proliferation-promoting effects on AF cells [25]. However, the molecular mechanisms underlying the therapeutic effects of PT on IDD remain unclear. We conducted in vivo experiments and successfully constructed an IDD model of 24-month-old naturally aging mice based on a previous study [26]. Our in vivo HE and safranin O/green staining results showed that PT treatment suppressed the progression of IDD. To the best of our knowledge, few studies have reported effects of PT against IDD via the regulation of oxidative stress and inflammation in NPCs. Based on prior work, *Plastrum testudinis* extract could possess antioxidant properties and provide protection against 2,2-diphenyl-1-picrylhydrazyl (DPPH)-induced oxidation by scavenging free radicals [27]. Moreover, our previous study has confirmed that *Plastrum testudinis* could also protect rat spine against glucocorticoid-induced oxidation and osteoporosis [28]. To the best of our knowledge, this study is the first to verify that PT can suppress TBHP-induced oxidative stress and inflammation in NPCs in vitro, thus alleviating IDD.

Numerous studies confirm that the active components of PT are involved in eliciting the anti-oxidant effect. For example, phenylalanine is a significant and efficacious amino acid in alleviating the adverse impacts of oxidative damage [29]. An existing study confirmed that plasma phenylalanine participated in regulating the expression of Col1a1 [30]. Threonine has been reported to take part in alleviating cadmium-induced oxidative stress [31]. The mitochondrial-targeted serine/threonine kinase PINK1 played as a protective role in clearance of damaged mitochondrial and alleviating cell senescence under oxidative stress in IDD [32]. Aspartic acid is reported to reduce oxidative stress and mitochondrial dysfunctions [33]. Reduced leucine concentration regulates mTORC1 pathway, thus reducing oxidative stress [34].

The 32 overlapping target proteins identified in this study were implicated in oxidative stress, inflammation, and tissue remodeling. GO enrichment analysis revealed terms related to oxidative stress and inflammation. This suggests that the identified proteins have essential roles in the mechanism underlying the effect of PT treatment on IDD. TBHP was used to induce oxidative stress and inflammation in NPCs in our in vitro experiments, where these processes contribute to the pathogenesis of IDD [35,36]. We detected intracellular ROS in TBHP-induced NPCs using an ROS probe DCFH-DA, confirming the protective effect of PT against oxidative stress (which induces senescence of NPCs) [37]. Existing evidence revealed that TNF-α-activated senescence in human NPCs could be attenuated through the PI3K/Akt pathway [38]. SA-β-Gal activity assessment showed that the administration of PT significantly attenuated NPC senescence. PT (0.5–2.5 μg/mL) significantly downregulated protein expression of the cellular senescence marker p16. Our study demonstrated the protective effects of PT against oxidative stress-induced damage in NPCs, suggesting that PT is a promising antioxidant for relieving oxidative stress in NPCs, and thus may improve IDD. Additionally, the TNF-α signaling pathway had the highest *p*-value in the KEGG enrichment analysis; PTGS2, EDN1, TNF-α, JUN and FOS were enriched in this pathway, and may be essential for the therapeutic effects of PT against IDD.

TNF-α is an important cytokine with strong proinflammatory activity; its expression is upregulated in IDD, and is closely associated with various pathological processes therein, including oxidative stress, the inflammatory response, cellular senescence, and apoptosis [11]. TNF-α can induce oxidative damage in NPCs, eventually resulting in IDD [39]. Additionally, TNF-α stimulation reduced the expression of COL2A1 and ACAN in NPCs [10]. Our study revealed that PT can reduce TNF-α expression in NPCs treated with TBHP, suggesting that PT may reduce the oxidative damage in NPCs seen in IDD patients by downregulating TNF-α expression. In addition, using LPS as an agonist, we verified that the protective effect of PT involves blockade of the TNF-α signaling pathway.

FOS is a transcriptional product encoded by the *C-FOS* gene, and has an important regulatory role in the cell cycle [40]. FOS is a vital signaling intermediate in NPCs that could be modulated by MAPK and PKC pathway activity in NPCs [41]. The inhibition of FOS expression in NPCs suppresses the expression of MMPs and inflammatory cytokines that promote the progression of IDD, and consequently has therapeutic effects with respect to IDD and associated pain [42]. JUN is a proinflammatory factor that forms AP-1 subunits, along with FOS [43,44]. The inflammatory response of NPCs is mediated by the AP-1-dependent activation of FOS and JUN [45]. In addition, attenuation of the phosphorylation of the AP-1 subunits of FOS/JUN in NPCs suppresses the inflammatory response associated with IDD [46]. Upregulation of FOS and JUN in Wnt3A-stimulated chondrocytes reduces the expression of COL2A1 and ACAN [47]. Our results verified that PT decreases the expression of AP-1 subunits of FOS/JUN in NPCs treated with TBHP, indicating that PT could reduce the inflammatory response in NPCs and thus ameliorate IDD.

PTGS2, also called COX-2, plays a vital role in the pathogenesis of the inflammatory response [48]. PTGS2 is an inflammatory factor in NPCs involved in the pathogenesis of IDD [49]. PTGS2 expression is increased by the activation of FOS and JUN in NPCs [45]. Suppression of PTGS2 in NPCs increased COL2A1 and ACAN levels [50]. In our study, TBHP-treated NPCs showed increased expression of PTGS2, as reported previously [36]. Additionally, PT significantly reduced the TBHP-stimulated increase in PTGS2 gene expression, indicating that PT could decrease the inflammatory response of NPCs by suppressing the expression of PTGS2 in IDD patients, thus exerting a therapeutic effect on IDD.

EDN1 is an inflammation modulator implicated in the degeneration of the cartilage end plate of the intervertebral disc; it is widely expressed in both non-vascular and vascular tissues [51]. EDN1 plays an active role in extracellular matrix (ECM) formation in cartilage [52]. Stimulation of EDN1 activates the Wnt/β-catenin signaling pathway and suppresses COL2A1, ACAN and SOX9 expression in cartilage end plate cells from degenerated discs, which leads to IDD [51]. Our results demonstrated that PT can decrease the expression of EDN1 in NPCs treated with TBHP, and thus may reduce the inflammatory response of NPCs by suppressing the expression of EDN1 in IDD patients.

COL2A1 (type II collagen) and ACAN (aggrecan) serve as NPC-specific markers, and play important regulatory roles in the synthesis of ECM and prevention of IDD [53]. The upregulation of TNF-α, FOS, JUN, PTGS2 and EDN1 suppresses the expression of COL2A1 and ACAN [10,47,50,51]. Therefore, we analyzed the expression of COL2A1 and ACAN, in NPCs. TBHP upregulated the expression of TNF-α, FOS, JUN, PTGS2 and EDN1, but downregulated the expression of COL2A1 and ACAN in NPCs. Treatment with PT had the opposite effects on NPCs treated with TBHP, which were exerted in a concentration-dependent manner.

In this study, the TNF-α signaling pathway had the highest *p*-value (Table 2), indicating that PT may exert protective effects against IDD by regulating this pathway. Inhibition of the TNF-α signaling pathway attenuates IDD progression by reducing inflammation and oxidative stress [54]. We showed that inflammation-specific genes, including TNF-α, FOS, JUN, PTGS2 and EDN1, are enriched in the TNF signaling pathway. We also confirmed that PT can reduce the expression of these elements of the TNF-α signaling pathway, while increasing that of COL2A1 and ACAN in NPCs treated with TBHP.

The pathway analysis also identified other signaling pathways that deserve further study. For example, the IL-17 signaling pathway exerts a proinflammatory effect in IDD [55]. IL-17 increases PGE_2_ production and COX-2 expression via the FOS and JUN subunits in NPCs, thus mediating inflammation of the intervertebral disc [45], and promotes IDD by suppressing autophagy through activation of the PI3K/Akt/Bcl-2 signaling pathway [56]. In addition, MAPK signaling pathway activity is an important mediator of NLRP3 inflammasome activity in NPCs [57]. Inhibition of the MAPK signaling pathway could ameliorate NPC senescence, thus suppressing IDD [58].

**Figure 7:**
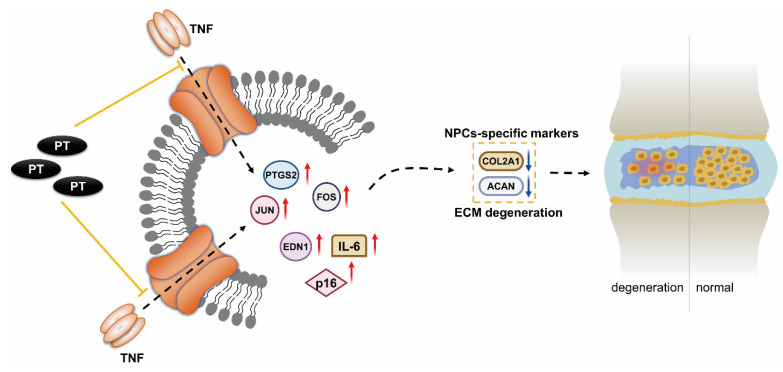
**Mechanism underlying the therapeutic effect of PT on IDD**. PT could downregulate the expression of inflammation-specific targets including TNF-α, FOS, JUN, PTGS2 and EDN1 on the TNF-α signaling pathway as well as cellular senescence marker p16, inflammatory factor IL-6, and upregulate the expressions of COL2A1 and ACAN, thus leading to a suppressive effect on inflammatory response and oxidative stress in NPCs.

Our results suggest that PT can suppress the expression of TNF-α, FOS, JUN, PTGS2, EDN1, IL-6, and p16, and upregulate that of COL2A1 and ACAN, thus reducing oxidative stress and inflammation in NPCs (Figure 7). Our results predict some potential therapeutic targets and pathways, providing reference for future studies on PT treatment against IDD. Further in vivo and in vitro experiments are needed to confirm our findings. Moreover, clinical studies on the safety and efficacy of this drug are scarce. Further research is required to confirm its clinical potential for the diagnosis and treatment of IDD. We plan to carry out such research in the future.

## 4. Materials and Methods

### 4.1. Retrieval of PT-Associated Components and Targets

We retrieved information on PT-associated bioactive components and targets by searching the BATMAN-TCM database (http://bionet.ncpsb.org/batman-tcm/, accessed on 31 March 2021), with a score cutoff of 10. The search was restricted to human organisms [59].

### 4.2. Retrieval of IDD-Related Genes

The human genetic database GeneCards (https://www.genecards.org/, accessed on 31 March 2021), which contains more than 190 data sources on diseases, genes, pathways, and components [60], was searched using the term “intervertebral disc degeneration”, with the species set as “*Homo sapiens*”.

### 4.3. Overlapping Target Proteins (OTPs)

We used R software (v3.6.1; R Development Core Team, Vienna, Austria) to determine the intersection of PT- and IDD-associated targets and obtain OTPs.

### 4.4. Protein–Protein Interaction Analysis of OTPs

To identify the relationships among the intersection targets, we searched the STRING database (https://string-db.org/, accessed on 31 March 2021) [61] for a protein–protein interaction (PPI) network related to OTPs, with the species limited to *Homo sapiens*.

### 4.5. GO and KEGG Analysis

Gene Ontology (GO) and Kyoto Encyclopedia of Genes and Genomes (KEGG) analyses of OTPs were conducted using the Cluster Profiler R package. We calculated the *p*-values of the corresponding enrichment results. Enrichment results significant at *p* < 0.05 were selected, and we plotted the pathway–target network using Cytoscape (http://www.cytoscape.org/, accessed on 31 March 2021).

### 4.6. Experimental Verification In Vivo and In Vitro

#### 4.6.1. IDD Model Establishment and Drug Intervention

**Figure 8:**
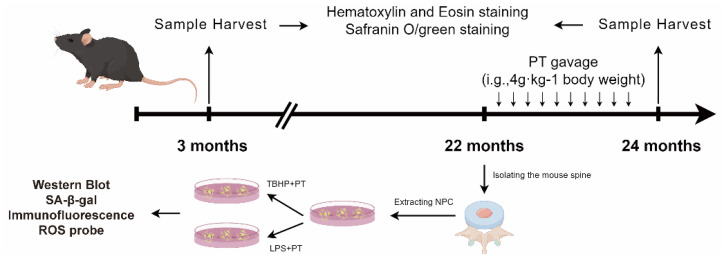
The schematic diagram to summarize the experimental design.

This research was approved by the Ethics Committee of our hospital (No. TCMF1-2019030), following the experimental designs (Figure 8). We bought randomly divided 3-month-old wild type C57BL/6 mice into three groups: control (CTL), IDD, and IDD with PT (IDD+PT). The animals were kept in a sterile environment with consistent light (12 h/day), temperature (21~26 °C), and humidity (41~70%) conditions, and were provided with adequate water and food. After 22 months, we obtained an IDD model of naturally aging mice according to a previous report [26].

The PT was purchased from our hospital (batch number KG37243537), and prepared based on a previously described method [23]. Briefly, we took 100 g of crushed PT, added 1 L of pure water, and boiled it gently for 1 h to obtain its extract. We added 800 mL of pure water to the remaining residue, boiled it gently for 1 h, took the extract, and repeated this process once more. To prepare drug treatment, the extract from PT was concentrated to 500 mL by a rotary evaporator heated to 60 °C. The CTL and IDD groups were treated with equal amount of solvent PBS, while the IDD+PT group was treated with PT. The dose was equivalent to that of humans and in accordance with the body surface area ratio [62]. The drug was administered by gavage at a dose of 4 g/kg/d for 2 months.

#### 4.6.2. Histological Evaluation

Samples were fixed in 10% neutral-buffered formalin, decalcified in ethylenediaminetetraacetic acid (EDTA) solution (pH 7.4), dehydrated, and embedded in paraffin. Serial sections of 5 μm thickness were taken from the midsagittal region for histological analysis. We performed hematoxylin–eosin (HE) and safranin O/green staining of the slices for morphometric analysis to observe histological changes in the intervertebral disc tissues from the different groups. All slides were observed under the microscope (BX53, Olympus Corp., Tokyo, Japan). Photographs were analyzed with the aid of CellSens Dimension software (version 510-UMA-CellSens19.0-krishna-ch-00-01; Hamburg, Germany). The histological assessment of IDD was based on a modified Thompson grading scale for nucleus pulposus cells (NPCs) and AF cells, as reported previously [63,64].

#### 4.6.3. NPC Culture and Treatment

We obtained nucleus pulposus tissues from the IDD model via digestion of 0.25% trypsin (Gibco, Waltham, MA, USA) for half an hour and 0.25% type II collagenase (Sigma, St. Louis, MO, USA) for 8 h at 37 °C. After centrifugation, NPCs were harvested and incubated in complete DMEM/F12 medium with 1% penicillin-streptomycin and 15% fetal bovine serum, under hypoxic conditions (5% CO_2_ in a humidified incubator at 37 °C). Second-generation NPCs were collected for subsequent experiments to prevent their differentiation [65].

#### 4.6.4. Cell Counting Kit-8 Assay

The NPCs were treated with tert-butyl hydrogen peroxide (TBHP; concentration gradient = 0, 50, 100, 150, 200, and 250 μmol/L) for 2, 4, and 6 h, and 5 μg/mL of PT (concentration gradient = 0, 0.5, 1, 2.5, and 5 μg/mL) for 24 and 48 h. The Cell Counting Kit-8 (CCK-8) (GK10001) was bought from GlpBio. Then, CCK-8 assay was used to detect cell viability in different groups.

#### 4.6.5. Western Blot Analysis

The Reagents including β-actin (AF7018), TNF-α (AF7014), and p16 (AF0228) antibodies were obtained from Affinity Biosciences (Cincinnati, OH, USA). ACAN (sc-166951) and COL2A1 (sc-52658) were from Santa Cruz (Shanghai, China), and prostaglandin G/H synthase 2 (PTGS2) (WL01750), endothelin-1 (EDN1) (WL02780), JUN (WL02863), FOS (WL03699), and IL-6 (WL02841) were from Wanleibio Co. Ltd. (Shenyang, China). We diluted the primary antibody with QuickBlock^TM^ Primary Antibody Dilution Buffer for Western Blotting (Beyotime, Shanghai, China) at a ratio of 1:1000, and the secondary antibody with QuickBlock^TM^ Secondary Antibody Dilution Buffer for Western Blotting (Beyotime) at a ratio of 1:10,000.

After TBHP treatment, with or without PT in a concentration gradient, mouse NPCs were seeded in 100 mm culture dishes (2 × 10^6^ cells). Then, protein extracts from cells were lysed in 200 μL RIPA lysis buffer (prepared with phosphatase inhibitor and protease inhibitor; Beyotime). The protein bands were transferred to polyvinylidene fluoride membranes (microtiter wells; Beyotime) via electrophoresis and wet transfer, and closed with QuickBlock^TM^ (Beyotime) at room temperature over 30 min. Primary antibodies were added and the solution was incubated overnight at 4 °C in a shaker; then, the corresponding secondary antibody was added and the solution was incubated in a shaker at 24 °C for 1.5 h. The antibody reactivity level was subsequently detected by a gel imaging system (Bio-Rad Laboratories, Hercules, CA, USA). Finally, the grayscale values were calculated using ImageJ software (Software Version: v2.1.4.7; NIH, Bethesda, MD, USA).

#### 4.6.6. Senescence-Associated β-Galactosidase (SA-β-Gal) Assay

After treatment with TBHP, with or without PT in a concentration gradient, mouse NPCs were washed once with phosphate-buffered saline (PBS); 1 mL of fixative solution (2% glutaraldehyde and 2% formaldehyde) was added, and the solution was fixed at room temperature for 15 min, stained with 1 mL β-galactosidase staining solution, and incubated overnight at 37 °C. Then, we acquired and analyzed images under a microscope, as reported previously [66].

#### 4.6.7. Cell Immunofluorescence Staining

NPCs were cultured in 24-well plates (4 × 10^4^ cells/well) and fixed for 15–20 min with 4% paraformaldehyde. After washing with PBS containing 0.1% Tween-20 (PBST), the samples were incubated with 0.2% Triton X-100 for 15 min and then blocked with QuickBlock^TM^ Blocking Buffer for Immunol Staining (P0260) for 30–60 min. The cells were treated with primary antibodies against ACAN (1:100) and COL2A1 (1:100) overnight at 4 °C. Secondary antibodies Goat Anti-Mouse IgG H&L (Alexa Fluor® 488) (ab150113) coupled with fluorescein isothiocyanate were then incubated at 37 °C for 1 h. Fluorescence images were obtained by fluorescence microscopy (Leica Microsystems, Inc., Buffalo Grove, IL, USA).

#### 4.6.8. Measurement of Intracellular ROS

A ROS probe called 2,7-dichloro-dihydro-fluorescein diacetate (DCFH-DA) was employed for detection of intracellular ROS in NPCs. Therefore, we purchased an ROS probe (S0033S) from Beyotime. First, NPCs were seeded onto 24-well plates and incubated for 24 h. After the interventions of TBHP with or without PT in a concentration gradient, they were treated with bupivacaine and the levels of intracellular ROS were evaluated by DCFH-DA. NPCs were washed twice in PBS followed by staining using 20 μM of DCFH-DA, for 30 min in darkness. Next, fluorescence was determined at an excitation wavelength of 485 nm and emission wavelength of 530 nm employing a fluorescence spectrometer (HTS 7000; PerkinElmer, Waltham, MA, USA). To analyze the pixel intensity of ROS quantification, ImageJ software was used to obtain fluorescence intensity in every group. We calculated the mean gray values of three measurements, which were used to plot a histogram to show the ROS levels in different group.

#### 4.6.9. Statistical Analyses

GraphPad Prism 8 (GraphPad Software Inc., La Jolla, CA, USA) was used for the statistical analyses. All data are expressed as the mean ± standard deviation. Student’s *t*-test was used to compare two samples, and a modified Thompson grading scale was used for analyses of the NPCs and AF cells along with the χ2 test.

## 5. Conclusions

The research into the antioxidant effects of PT has yielded important findings. In this study, utilizing IDD model mice, PT demonstrated a therapeutic effect on IDD by regulating oxidative stress and inflammation. Notably, the observed effects of PT were associated with downregulation of the TNF-α signaling pathway.

This suggests a potential therapeutic value of PT in mitigating IDD in model animals. While these findings hold promise, further research is needed to elucidate the precise molecular mechanisms of PT’s antioxidant action, and explore potential interactions with existing antioxidant treatments.

## Figures and Tables

**Table 1:** Potential target genes for PT in the treatment of IDD.

Number	Gene	Number	Gene
1	PTGS1	17	CAV1
2	PTGS2	18	C1QTNF3
3	EDN1	19	PTK2
4	NOS2	20	TAT
5	IL2	21	PAH
6	TNF	22	DDAH1
7	JUN	23	ATF3
8	FOS	24	SLC6A4
9	PAICS	25	HGD
10	SUCLG1	26	GSTZ1
11	PLOD1	27	HPD
12	SDHA	28	DBH
13	SUCLA2	29	BDNF
14	CACNA1A	30	FAH
15	GRIN2A	31	TRPV1
16	COMT	32	PRODH

**Table 2:** KEGG pathway enrichment results.

ID	Signaling Pathway	Enriched Genes	*p* Value
hsa04668	TNF signaling pathway	PTGS2/EDN1/TNF/JUN/FOS	0.000042
hsa04657	IL-17 signaling pathway	PTGS2/TNF/JUN/FOS	0.000324
hsa04625	C-type lectin receptor signaling pathway	PTGS2/IL2/TNF/JUN	0.000476
hsa04660	T cell receptor signaling pathway	IL2/TNF/JUN/FOS	0.000476
hsa04024	cAMP signaling pathway	GRIN2A/EDN1/JUN/FOS/BDNF	0.000966
hsa04926	Relaxin signaling pathway	EDN1/NOS2/JUN/FOS	0.001072
hsa04010	MAPK signaling pathway	CACNA1A/TNF/JUN/FOS/BDNF	0.003534
hsa04933	AGE-RAGE signaling pathway	EDN1/TNF/JUN	0.005289
hsa04620	Toll-like receptor signaling pathway	TNF/JUN/FOS	0.005900
hsa05022	Pathways of neurodegeneration	SDHA/GRIN2A/PTGS2/NOS2/TNF/BDNF	0.005959

## Data Availability

Data is contained within the article.

## Abbreviations

PT	*plastrum testudinis*
IDD	intervertebral disc degeneration
PTGS2	prostaglandin G/H synthase 2
EDN1	endothelin-1
SA-β-gal	senescence-associated β-galactosidase
NPC	nucleus pulposus cell
TNF	tumor necrosis factor
MMPs	matrix metalloproteinases
ROS	reactive oxygen species
AF	annulus fibrosus
LPS	lipopolysaccharide
PPI	protein–protein interaction
GO	Gene Ontology
KEGG	Kyoto Encyclopedia of Genes and Genomes
HE	hematoxylin–eosin
BATMAN-TCM	Bioinformatics Analysis Tool for Molecular Mechanism of Traditional Chinese Medicine
STRING	Search Tool for the Retrieval of Interacting Genes/Proteins
CTL	control
TBHP	tert-butyl hydrogen peroxide
DCFH-DA	2,7-dichloro-dihydro-fluorescein diacetate
PBS	phosphate-buffered saline
RIPA	radioimmunoprecipitation assay
BP	biological process
AP-1	activator protein 1
PTGS2	prostaglandin G/H synthase 2
COX-2	cyclooxygenase-2
EDN1	endothelin-1
COL2A1	type II collagen
ACAN	aggrecan
ECM	extracellular matrix
